# Health benefits of microalgae and their microbiomes

**DOI:** 10.1111/1751-7915.14082

**Published:** 2022-05-29

**Authors:** Ines Krohn, Simon Menanteau‐Ledouble, Gunhild Hageskal, Yekaterina Astafyeva, Pierre Jouannais, Jeppe Lund Nielsen, Massimo Pizzol, Alexander Wentzel, Wolfgang R. Streit

**Affiliations:** ^1^ Department of Microbiology and Biotechnology University of Hamburg Hamburg Germany; ^2^ Department for Chemistry and Bioscience Aalborg University Aalborg Denmark; ^3^ Department of Biotechnology and Nanomedicine SINTEF Industry Trondheim Norway; ^4^ Department of Planning Aalborg University Aalborg Denmark

## Abstract

Microalgae comprise a phylogenetically very diverse group of photosynthetic unicellular pro‐ and eukaryotic organisms growing in marine and other aquatic environments. While they are well explored for the generation of biofuels, their potential as a source of antimicrobial and prebiotic substances have recently received increasing interest. Within this framework, microalgae may offer solutions to the societal challenge we face, concerning the lack of antibiotics treating the growing level of antimicrobial resistant bacteria and fungi in clinical settings. While the vast majority of microalgae and their associated microbiota remain unstudied, they may be a fascinating and rewarding source for novel and more sustainable antimicrobials and alternative molecules and compounds. In this review, we present an overview of the current knowledge on health benefits of microalgae and their associated microbiota. Finally, we describe remaining issues and limitation, and suggest several promising research potentials that should be given attention.

## Graphical abstract

Microalgae may offer solutions to the societal challenge we face concerning the lack of antibiotics treating the growing level of antimicrobial resistant bacteria and fungi in clinical and industrial settings. While the vast majority of microalgae and their associated microbiota are unstudied, they may be a fascinating and rewarding source for novel and more sustainable antimicrobials and alternative molecules and compounds. In this review we present an overview of the current knowledge on health benefits of microalgae and their microbiota.

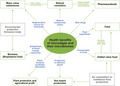

## Introduction and background

Microalgae and their associated microbiota grow and survive in all climate zones and many species are well adapted to extreme temperatures and pH values. Since microalgae are photosynthetic active organisms, which can be grown under a wide variety of conditions, they are highly attractive for the biotechnological production of a wide range of different chemical compounds. They are particularly well known for their use in the production of advanced biofuels (e.g. drop‐in biofuels and fourth‐generation biofuels) and to some extend for the production of bioplastics (Chisti, 2007; Mata *et al*., 2010; Hempel *et al*., 2011; Rahman and Miller, 2017; Khan *et al*., 2018; Onen Cinar *et al*., 2020; Keasling *et al*., 2021).

**Fig. 1:**
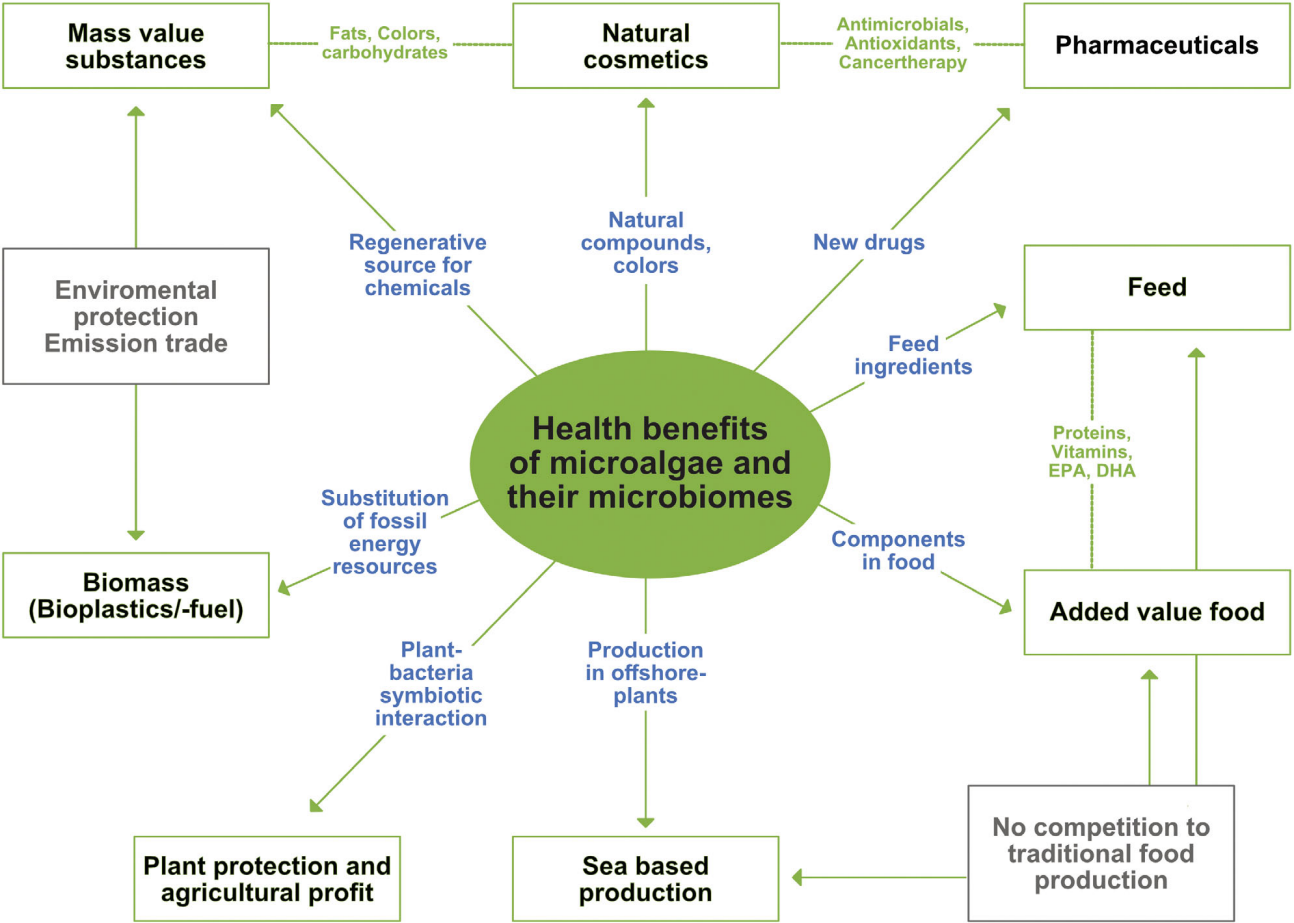
Applications for microalgae including their microbiomes. Overview of potential microalgae and their communities' production and application fields; mainly reflecting clinical and human health, animal health and plant protection.

Recently, it has become clear that algae and their microbiota harbour a large and diverse set of genes for the biosynthesis of molecules that supress bacterial pathogens (Table 1) (Krohn‐Molt *et al*., 2013, 2017). One of the example are sterols with anti‐inflammatory capacity, like diacylglycerols, triacylglycerols and phytosterols (Ostlund *et al*., 2003; Bilbao *et al*., 2016; Randhir *et al*., 2020). In this respect, they bear a great potential to make major contributions to important societal needs linked to the treatment of infections due to human, animal and plant pathogenic microorganisms (Fig. 1). The appearance of untreatable antibiotic resistant microorganisms in clinical settings is a major concern to human health (O'Neill, 2016; WHO, 2019). Thus, there is a need to develop novel antimicrobials that are distinct in their mode of action from those already known and on the market. The awareness in the scientific community about the largely unexplored potential in microalgae has led to increased interest during the last decade, as evidenced by an exponential increase in the number of publications and patents on the subject of microalgae and health. However, compared to the large number of microalgal species, our knowledge remains sparse, and further research requires a focused and more systematic approach to better explore this promising resource with a special emphasis on human, animal and plant health and well‐being. Here, we summarize current knowledge on the benefits of microalgae in health management. We further point out current limitations hindering their exploitation and address technologies that could provide a basis for a more systematic exploitation of their potential.

**Table 1:** Key features and bioinformatical analysis of microalgae genomes and metagenomes.

Key features and bioinformatical analysis of microalgae genomes and metagenomes	*Chlamy‐domonas reinhardii*	*Arthro‐spira platensis*	*Oscilla‐toria acuminata* PCC 6304	*Gloeo‐capsa* sp.	*Chryso‐chromu‐lina tobin*	*Chlorella variabilis* NC64A	*Cocco‐myxa subellip‐soidea* C‐169	*Oceani‐caulis* sp. HL‐87 GFM and their micro‐biome	*Lyngbya* sp. HA4199‐MV5 and their micro‐biome	*Scene‐desmus quadri‐cauda a*nd their micro‐biome	*Micras‐terias crux‐melitensis* and their micro‐biome	*Chlorella saccharo‐phila* and their micro‐biome	*Chlorella soro‐kiniana* and their micro‐biome
IMG ID	2614208541	650377906	2509276028	2503754017	3300021056	2507525016	2507525016	2588254262	3300034630	3300005759	3300008886	3300008885	3300042370
Size (bp)	111100715	6788435	7804270	5882710	101136936	2758551	2758551	2758551	56655633	168690013	268162588	174773623	2758551
Antibacterial activity													
Dienelactone hydrolase	6	4	3	6	40	2	2	3	52	75	255	100	60
Imidazolone‐propionase	1	0	0	0	23	1	0	5	42	57	205	97	37
6‐phosphogluco‐nolactonase	2	1	2	1	21	3	1	0	26	36	115	54	16
Metal‐dependent hydrolases, COG1235	0	3	3	0	14	0	0	1	13	23	96	34	14
Sugar lactone lactonase YvrE	0	0	1	2	24	3	3	1	43	74	202	113	30
Decanoic acid, capric acid, decylic acid (tetradecanoate)	5	4	5	6	0	3	5	5	0	0	0	0	0
Palmitoleic acid (palmitoleate)	12	8	8	10	0	13	11	8	151	0	0	0	0
gamma‐Linolenic acid, γ‐linolenic acid (γ‐linolenate)	4	2	2	2	0	4	5	1	28	0	0	0	0
Arachidonic acid, polyunsaturated omega‐6 fatty acid (arachidonate)	17	0	0	0	0	22	38	0	0	0	0	0	0
Docosahexaenoic acid (DHA)	18	1	1	0	0	40	32	6	72	0	0	0	0
Eicosapentaenoic acid (EPA) (docosapentae‐noate)	13	1	1	0	0	31	49	6	47	0	0	0	0
Antiviral activity													
Phycoerythrobilin biosynthesis	2	2	4	2	0	6	4	0	2	0	0	0	0
Phycocyanobilin biosynthesis	1	2	3	2	0	4	3	0	1	0	0	0	0
Phycoviolobilin biosynthesis	1	2	3	2	0	4	3	0	1	0	0	0	0
Phycourobilin biosynthesis	1	1	2	1	0	3	2	0	2	0	0	0	0
Exopolysaccharide biosynthesis	0	3	1	6	22	1	0	2	31	54	121	55	32
D‐galactose biosynthesis	1	1	2	5	18	2	1	0	21	43	199	97	29
L‐arabinose biosynthesis	0	0	0	0	4	0	1	0	2	1	20	4	3
D‐xylose biosynthesis	1	1	2	1	2	1	1	0	4		37	13	4
L‐rhamnose biosynthesis	0	6	7	8	34	3	2	4	45	64	282	146	55
D‐galacturonate biosynthesis (D‐galacturonic acid)	1	1	1	1	7	0	0	1	10	12	52	32	8
Mannose biosynthesis	4	6	6	6	30	4	7	2	39	63	290	144	51
Fucose biosynthesis	0	4	4	3	35	1	1	2	38	66	264	135	53
Antioxidant activity													
Superoxide dismutase	6	0	2	2	15	4	6	1	18	22	90	46	20
Cu/Zn superoxide dismutase	0	0	0	0	6	0	0	0	6	8	23	9	4
Rhodanese‐related sulfurtransferase	11	3	4	5	44	10	8	0	43	53	118	55	57
Catalase (peroxidase I)	1	0	0	0	15	0	0	1	14	28	86	44	16
Catalase	1	0	0	2	18	1	4	0	13	19	51	30	10
Mn‐containing catalase (includes spore coat protein CotJC)	0	0	1	2	0	0	0	0	2	6	8	3	4
Ferritin, oxidative damage protectant	6	2	3	5	8	0	0	4	67	18	61	28	13
Glutaredoxin	8	4	3	4	41	13	8	4	50	59	188	112	38
Glutathione peroxidase	4	0	0	0	27	6	3	0	12	18	64	29	18
Cytochrome c peroxidase	0	1	0	1	4	0	0	0	6	17	68	21	15
Alkylhydroperoxidase	0	0	0	1	13	0	0	1	69	72	276	138	64
Deferrochelatase/peroxidase	0	0	4	0	1	0	0	0	5	11	41	26	
Peroxiredoxin	9	11	12	8	54	9	11	6	54	78	314	139	74
Chlorophyll a biosynthesis	5	4	4	4	0	6	6	0	5	0	0	0	0
Carotenoid biosynthesis	0	0	2	0	0	13	13	0	0	0	0	0	0
Lutein biosynthesis	2	2	0	3	0	2	3	1	6	0	0	0	0
Zeaxanthin epoxidase	0	0	0	0	0	1	1	0	0	0	0	0	0
Violaxanthin de‐epoxidase	0	0	0	0	0	1	1	0	0	0	0	0	0
astaxanthin biosynthesis	0	0	0	2	0	1	1	0	8	0	0	0	0
Anti‐inflammatory and anti‐cancer properties													
Phytosterol biosynthesis	5	0	0	0	0	5	7	0	1	0	0	0	0
Zymosterol biosynthesis	1	0	0	0	0	1	1	0	0	0	0	0	0
Ergosterol biosynthesis	7	0	0	0	0	7	7	0	2	0	0	0	0
Cholesterol biosynthesis	19	0	0	0	0	19	24	0	3	0	0	0	0
Sulfoquinovosyl diacylglycerol biosynthesis	1	1	1	1	1	1	1	0	4	1	0	1	1
Diacylglycerol and triacylglycerol biosynthesis	10	3	2	2	40	14	14	3	43	65	254	125	48
Immune promoters and immunomodulatory activity													
1,4‐alpha‐glucan branching enzyme	4	2	3	3	0	2	2	0	12	0	0	0	0
Bacterial‐like globin (possible phycocyanins)	11	0	1	1	0	5	2	1	15	0	0	0	0
Carotenoid cleavage dioxygenase or a related enzyme	5	1	2	2	0	5	6	1	12	0	0	0	0
Bacterial lipopolysaccharides biosynthesis (LPS)	3	5	6	10	21	0	0	2	54	28	33	13	8
Prebiotic activity													
Beta‐1,3‐glucan (paramylon) synthase	0	0	0	0	0	4	1	0	0	0	0	0	0
mycolyl‐arabinogalactan‐peptidoglycan complex biosynthesis	1	3	5	9	0	2	1	2	22	0	0	0	0
Cellulose biosynthesis	0	1	6	3	0	2	8	4	14	0	0	0	0
Algin biosynthesis (GDP‐mannose biosynthesis)	4	6	6	6	0	4	7	2	39	0	0	0	0
GDP‐L‐fucose biosynthesis	0	2	2	1	0	0	1	0	17	0	0	0	0
dTDP‐3‐acetamido‐α‐D‐fucose biosynthesis	0	0	0	0	0	1	0	0	0	0	0	0	0
agar; carrageenans biosynthesis (β‐D‐; α‐D‐; α‐L‐galactose)	2	1	2	5	0	3	2	0	21	0	0	0	0
GDP‐L‐fucose synthetase	0	2	0	1	0	0	1	0	0	0	0	0	0
Beta‐galactosidase	2	0	1	2	0	2	11	0	16	0	0	0	0

Key features of antibacterial, antiviral, antioxidant activity and anti‐inflammatory and anti‐cancer properties as well as immune promoters and immunomodulatory activity of microalgae communities' genomes and metagenomes using IMG function search including IMG ID and total size of bp. Data shown in total number of hits for possible antibacterial activity, antiviral activity, antioxidant activity, anti‐inflammatory and anti‐cancer properties, and immune promoters and immunomodulatory activity.

## Antibacterial activity

### Quorum sensing and quorum quenching as drives for antibiofilm strategies

Quorum sensing (QS) and Quorum Quenching (QQ) play an important role in the expression of virulence factors and antimicrobial resistance, and are involved in the formation of bacterial biofilms (Ahlgren *et al*., 2011; Waters and Goldberg, 2019). The latter are of major concern in clinical and industrial settings, as they are very difficult to control and treat and cause severe problems in patients and industries. Both QQ and various mechanisms of QS interference have been outlined and discussed as possible strategies to prevent and treat microbial biofilm formation (Singh *et al*., 2000; Ahlgren *et al*., 2011; Fetzner, 2015; Waters and Goldberg, 2019). Microalgae and their associated bacterial microbiota may be valuable tools to further verify this concept (Table 1).

Microalgae microbiomes offer both QQ enzymes and a broad variety of QS molecules that have been shown to interfere with pathogens (Ghanei‐Motlagh *et al*., 2021a, 2021b). In this framework, the screening of 19 strains of microalgae, reported that one microbial community of *Chlorella saccharophila* and one of *Chlorella vulgaris* both degraded *N*‐acyl homoserine lactones (AHLs), resulting in the inhibition of violacein production in the reporter strain (Natrah *et al*., 2011). When using a *E. coli* (JB523)‐strain sensitive to N‐(3‐oxohexanoyl)‐L‐homoserine lactone, the *C. saccharophila* associated microbiome was found to significantly suppress bacterial QS and further to inhibit AHL‐regulated bioluminescence in the pathogen *Vibrio harveyi* (Natrah *et al*., 2011). AHL degradation can occur indirectly, such as in cultures of *Tetraselmis suecica* and *Chaetoceros muelleri* that were associated with AHL‐degrading bacteria belonging to the genera *Bacillus* and *Pseudomonas* (Pande *et al*., 2015). These bacterial isolates were found to degrade AHL molecules, and *Bacillus* sp. was reported to suppress the quorum sensing system of *Vibrio campbelli* and thus protected the larvae of the giant river prawn (*Macrobrachium rosenbergii*) from infection, improving survival from 42 to 67% during an infection challenge.

Another route of interference with quorum sensing systems is through the secretion of molecular mimics. In general, in Gram‐negative bacteria, many important changes in gene expression and behaviour are regulated in a population density‐dependent fashion by N‐acyl homoserine lactone (AHL) signal molecules. Plants are able to secrete substances, which mimic bacterial N‐acyl homoserine lactones. These mechanisms affect population density‐dependent behaviours in associated bacteria. For example, ethyl acetate, extracted AHL mimics from *Chlamydomonas reinhardii*, was found to affect the expression of 34 proteins, 25 of which were also affected by AHL (Teplitski *et al*., 2004).

The analysis of algae and microalga microbiomes available at IMG/MER (https://img.jgi.doe.gov) revealed numerous QQ genes. The study of metagenomes of *Scenedesmus quadricauda*, *Chlorella saccharophila*, *Chlorella sorokiniana* and *Micrasterias crux‐melitensis* unveiled dienelactone hydrolases, imidazolonepropionases, 6‐phosphogluconolactonases and metal‐dependent hydrolases, associated with QQ, which are potential candidates for overexpression experiments and biotechnological studies (Table 1).

### Phycobiliproteins have antimicrobial effects

Multiple compounds from microalgae and their affiliated microbiota have been reported to have antimicrobial properties, sometimes as a secondary benefit distinct from their primary function. This is the case for phycobiliproteins, a family of water‐soluble light‐harvesting pigments, that play a central role in the photosynthesis of cyanobacteria and the red algae *Rhodophyta*. Phycobiliproteins are divided into four groups: phycocyanin, phycoerythrin, phycoerythrocyanin and allophycocyanin, of which phycocyanin is the most common in the environment (Li *et al*., 2019, Pagels *et al*., 2019, Fig. 2). Overall, in our bioinformatic analyses, it was found that publicly available genomes of *Chlorella variabilis* NC64A and *Coccomyxa subellipsoidea* C‐169 (IMG ID 2507525016) contain phycoerythrobilin, phycocyanobilin, phycoviolobilin and phycourobilin biosynthesis genes (Table 1). For example, screening of 19 microalgae supernatants containing the whole microbial community identified multiple active phycobiliproteins, including phycocyanin and phycoerythrin, which exhibited significant antifungal property and growth inhibition of both Gram‐positive and ‐negative bacteria (Najdenski *et al*., 2013). A phycobiliprotein extract from *Arthrospira platensis* was found to have antifungal effect against the plant pathogen *Botrytis cinerea* when applied at doses from 0.3 to 4.8 mg ml^−1^, both reducing the fungal growth as well as protecting tomato fruits from infection (Righini *et al*., 2020). Interestingly, environmental conditions have been found to affect the production of phycobiliproteins, both in terms of quantity and of the distribution of phycobiliprotein produced, with factors such as the pH, quality of light and nutrient source all having a significant effect (Pagels *et al*., 2019). Khattar *et al*. (2015) reported optimized culture conditions for *Anabaena fertilissima* that involved a slightly alkaline pH as well as supplement of nitrite and illumination with blue light, resulting in a 1.6‐fold increase in the total production of phycobiliproteins (from 383 to 627 μg mg^−1^; *P* < 0.05) and a 4.5‐fold in the production of phycoerythrin (from slightly more than 100 to almost 500 μg mg^−1^; *P* < 0.05). Overall, as every microalgae will require its own optimization to achieve its maximum potential, it is one of the main issues for practical application of these products.

**Fig. 2:**
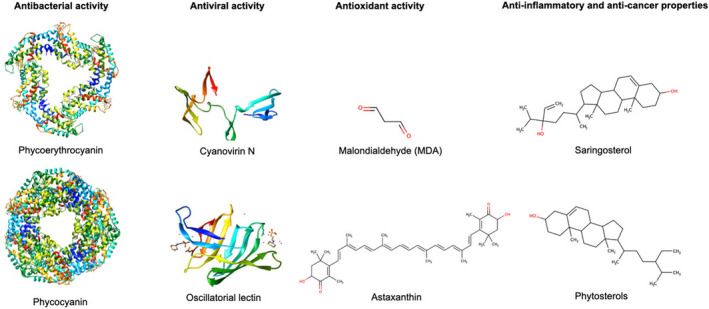
Molecular structures. Selected biologically active compounds derived from microalgae and their associated microbiota. Antibacterial activity: Phycocyanin (Wang *et al*., 2001), Phycoerythrocyanin (Schmidt *et al*., 2006), Antiviral activity: Cyanovirin N (Yang *et al*., 1999), Oscillatorial lectin (Koharudin *et al*., 2011), Antioxidant activity: Malondialdehyde (MDA), Astaxanthin (https://pubchem.ncbi.nlm.nih.gov/) Anti‐inflammatory and anti‐cancer properties: Saringosterol, Phytosterols (https://pubchem.ncbi.nlm.nih.gov/). Marvin was used for drawing, displaying and characterizing chemical structures, substructures and reactions, Marvin version 21.17.0, ChemAxon (https://www.chemaxon.com). UCSF Chimera was used for molecular modelling and for analysis of molecular structures, developed by the Resource for Biocomputing, Visualization and Informatics at the University of California, San Francisco, with support from NIH P41‐GM103311 (https://www.cgl.ucsf.edu/chimera/docs/credits.html).

### Fatty acids play a key role as antimicrobials

Fatty acids, including those from microalgae, have strong antimicrobial effects. Notably, extracts from cyanobacteria have been shown to be inhibitory against *Streptococcus pyogenes* and *Staphylococcus aureus*, while fatty acids from the cyanobacterium *Synechocystis* sp. were inhibitory against *Bacillus cereus*, *Escherichia coli* and the yeast *Candida albicans* (Najdenski *et al*., 2013). Ruffel et al. tested 29 different types of purified fatty acids from different species of microalgae using disk‐diffusion assay. The results show, that 3 of 29 fatty acids were inhibitory against *E. coli*, while 15 were inhibitory against *S. aureus* (Ruffell *et al*., 2016). The effective dose ranged from 250 to 2000 μg per disk and polyunsaturated fatty acids (PUFA) were found to be more significantly more likely to display antimicrobial activity compared to monounsaturated or saturated acids (11 of 13, compared to two of seven and two of nine respectively).

Testing of extracts from *Chlorococcum* strain HS‐IO1 and *Dunaliella primolecta* showed that α‐linolenic acid from these algae had antimicrobial properties against methicillin‐resistant *Staphylococcus aureus* (MRSA) (Ohta *et al*., 1995). Similarly, the fatty acid fraction of the acidophilic *Coccomyxa onubensis* has been shown to have antimicrobial activity against multiple Gram‐positive and ‐negative bacterial pathogens. Although these authors did not test the individual fatty acids involved in this activity, the most common fatty acids in the extracts included palmitic acid and oleic acid alongside the PUFA linoleic acid and linolenic acid (Navarro *et al*., 2017). In *Chlorella* spp., a mixture of fatty acids, termed ‘chlorellin’ is known to have antimicrobial properties, and, for example, ethanol and isopropanol‐extracts from *Chlorella* spp. were shown to have inhibitory capacity equivalent to ampicillin and oxacillin against *Staphylococcus* spp., although this author did not investigate the effect of the individual fatty acids (Acurio *et al*., 2018). Taken together, these results suggest that various microalgal fatty acids can exert antimicrobial activity, although PUFA appeared more likely to do so. Unfortunately, drawing firm conclusions is hindered by the fact that many authors treat all fatty acids together rather than attempt to separate the various species of fatty acids to test them separately.

Contrary to the percentage of lipids in microalgal cells which is considered roughly comparable between microalgal species, fatty acids are highly variable both in terms of their relative concentration and the repartition of the various species of fatty acids (Hu, 2013). Several environmental factors have been reported to influence the fatty acid profile of phytoplankton, for example, higher temperatures are associated with an increased in the proportion of saturated fatty acids, whereas decreased light levels were associated with an increased in polyunsaturated fatty acids (plausibly due to an increase in the presence of thylakoids to improve photosynthetic activity) (Guedes *et al*., 2010; Li *et al*., 2011). Interestingly, comparison of the fatty acid profiles of diatoms and dinoflagellates by Peltomaa *et al*. (2019) suggested that fatty acid contents were higher in freshwater species than marine ones. However, genetic and phylogeny appear to be the main factor dictating the fatty acid profile of microalgae. For example, screening of 1145 species, representing six major groups of both marine and freshwater phytoplankton species, showed that phylogeny was the main factor influencing the fatty acid profile of microalgae (Galloway and Winder, 2015) accounting for about 36 to 44% of the total variation in fatty acid profiles. Similar results were reported by Jónasdóttir (2019) based on the screening of the fatty acid profiles of 160 species representing seven phyla of marine phytoplankton.

For example, Cavonius *et al*. (2014) reported that fatty acid represented 12% of the dry mass of *Tetraselmis galbana*, but only 4–5% in *Phaeodactylum tricornutum*. Similarly, screening of 2076 strains of microalgae by Lang *et al*. (2011) showed that fatty acids were particularly low in *Chlorophyta* and *Streptophyta*. Comparison of the fatty acid profiles of six commonly cultivated strains of microalgae showed that *C. vulgaris* had a comparatively high concentration of 16:2 fatty acid. In addition, both *C. vulgaris* and *Tetradesmus obliquus* had comparatively high levels of 18:1 fatty acids as well as 18:3, linolenic acid (Chacón‐Lee and González‐Mariño, 2010). This is in accordance with the reports of Cepas *et al*. (2021) who, after screening the fatty acid profiles of several strains of cyanobacteria also reported that *C. vulgaris* and *T. obliquus* were particularly rich in linolenic acid.

### Other microalgal molecules with antibacterial effects

EPS from several algal cultures were inhibitory against multiple bacterial and fungal isolates when tested using both the agar diffusion and the minimal inhibitory concentration (MIC) (Najdenski *et al*., 2013). Of these, the most effective were EPS from *Gloeocapsa* sp. with MIC values ranging from 0.125 for *S. aureus* to 1.0 mg ml^−1^ for *S. pyogenes* (Najdenski *et al*., 2013). Similarly, crude extracts from *H. pluvialis* demonstrated inhibitory effects against several bacterial pathogens using disk‐diffusion assays, resulting in inhibition zone between 6.1 and 10.2 mm (Rather *et al*., 2021).

Extraction of *A. platensis* compounds using different solvents revealed that methanolic extracts had the highest antimicrobial activity against bacterial pathogens. Whereby, MIC of were 128 and 256 μg ml^−1^ against *S. aureus* and *E. coli*, respectively, although the compounds involved were not further characterized (Kaushik and Chauhan, 2008). Extraction of a variety of antimicrobial compounds from *Cosmarium* sp. showed that all had some potential as antimicrobials, although the methanol, hexane and aqueous extracts were not effective against the Gram‐positive bacteria tested (Challouf *et al*., 2012).

Other microalgae cultures including their microbiomes have been found to display antimicrobial properties, but without the responsible compounds being further characterized. This is the case for eight freshwater microalgae (belonging to the genera *Oscillatoria*, *Lyngbya*, *Oedogonium* and *Spirogyra*) whose ethanolic and methanolic fractions, tested at concentrations ranging from 0.16 to 0.66 mg ml^−1^ using the disk‐diffusion method, demonstrated some inhibitory properties against some Gram‐negative and one Gram‐positive pathogenic bacteria with zone of inhibitions ranging from 7 to 12 mm, for *O. sancta* extracted using ethanol at a dose of 0.35 mg ml^−1^ and *S. decimina* extracted using a methanol solvent and applied at 0.20 mg ml^−1^ respectively (Prakash *et al*., 2011).

For example, methanolic extracts from the cyanobacterium *A. platensis* at 100 ng ml^−1^ have clear inhibitory effects on the biofilm formation of several bacteria, including pathogens. Biofilm formation of *Vibrio parahaemolyticus* was inhibited by 90%, of *Vibrio alginolyticus* by 88%; of *Aeromonas hydrophila by* 74%; and by 61 to 84%, in *S. aureus* (LewisOscar *et al*., 2017).

In addition, for the green alga *Chlamydomonas reinhardtii*, Vishwakarma and Sirisha (2020) reported that extracted sulfated polysaccharides displayed activity against the biofilms of *Salmonella enterica* and *V. harveyi*, distorting the biofilms and reducing their formation by about 50% when applied at concentrations of 0.5 mg ml^−1^ against *S. enterica* and 8 mg ml^−1^ against *V. harveyi*. Ghaidaa *et al*. (2020) reported similar findings with *C. reinhardtii* reducing the formation of biofilms of several bacterial species by about 50%. Several human pathogens were also more susceptible to these compounds since *S. aureus* biofilms were reduced by up to 90%.

### Antiviral activity

In cyanobacteria, a variety of antiviral and antimicrobial molecules has been described over the years, as recently reviewed by Mazur‐Marzec *et al*. (2021) and Khalifa *et al*. (2021), including cyanovirin N, isolated from *Nostoc ellipsosporum*, which is known to interfere with human immunodeficiency virus (HIV's) binding onto CD+ T‐cells. The antiviral compound cyanovirin‐N binds the viral spike protein gp120 that is required for HIV interactions with receptors on the host cells and has been shown to have antiviral activity against HIV (Dey *et al*., 2000; Singh *et al*., 2005). Cyanovirin‐N has also demonstrated inhibitory action on other enveloped viruses such as herpes virus and measles virus as well as feline immunodeficiency virus (FIV) at concentrations as low as 10 nM (Dey *et al*., 2000). More recently, *in silico* docking simulations have suggested that cyanovirin‐N could form stable covalent bonds with the homotrimeric transmembrane spike glycoprotein of severe acute respiratory syndrome coronavirus‐2 (Lokhande *et al*., 2020).

Among these, lectins are ubiquitous proteins, whose carbohydrate domains have been shown to interact with the surface of cells and viruses. For example, a lectin recently isolated from the cyanobacterium *Oscillatoria acuminate* was found to be inhibitory against herpes simplex virus type‐1 at doses at low as 20 ng ml^−1^ with highest inhibition at a dose of 2 mg ml^−1^ (Saad *et al*., 2020). Because pre‐treating the cells prior to viral infection was not found to be protective, it is most likely that this lectin interacts and interferes with viral receptors. Figure 2 shows as an example the structures of cyanovirin N and oscillatorial lectin.

A further interesting fact is that many microalgae communities are able to produce polysaccharides, which are well known to exert a broad spectrum of biological activities, especially antiviral properties (Chaisuwan *et al*. 2021). These polysaccharides were found among numerous algal microbiomes, including *S. quadricauda* (IMG ID 3300005759), *C. saccharophila* (IMG ID 3300008885), *C. sorokiniana* (IMG ID 3300042370) and *M. crux‐melitensis* (IMG ID 3300008886) (Table 1).

### Antioxidant activity

It is well established that multiple compounds from microalgae have antioxidant activity. The best known algal antioxidant is the keto‐carotenoid pigment astaxanthin, in particular extracted from the green algae *Haematococcus pluvialis* (Plaza *et al*., 2009, Fig. 2). Astaxanthin can neutralize singlet oxygen and scavenge free radicals, resulting in a powerful antioxidant effect, approximately 10 times stronger than β‐carotene and 100 times greater than that of α‐tocopherol (Shimidzu *et al*., 1996; Lorenz and Cysewski, 2000). *H. pluvialis* exists under different morphotypes, influenced by environmental conditions, including high temperature, intense light, in particular UV‐light, alongside other stressors such as salinity, drought or nutrient scarcity. Under the effect of these environmental stressors, it switches from a flagellated free‐living cell to a coccoid cyst called an aplanospore (Lim *et al*., 2018; Molino *et al*., 2018). This is accompanied by a degeneration of the cell's chloroplasts, hence the name ‘red phase’ given to this phase of the life cycle, a thickening of the outer cell wall and secretion of two layers of extracellular matrix as well as the accumulation of large quantities of astaxanthin in oil droplets within the aplanospore (Wayama *et al*., 2013). During this process, the relative volume of astaxanthin changes from 0.2% of the total free‐living cell to 52% in the aplanospores.

The antioxidant capacity of astaxanthin has recently been shown in juvenile Asian tiger shrimp (*Penaeus monodon*). Supplementation of 80 mg astaxanthin per kg of diet was associated with a significant increase in the total antioxidant status and superoxide dismutase (SOD) in the haemolymph of the shrimp (Pan *et al*., 2003). This was connected to an improved recovery following exposure to various stressors and a decrease in both alanine aminotransferase (ALT) and aspartate aminotransferase (AST) values in the haemolymph, suggesting a hepatoprotective effect of astaxanthin. Comparable results were reported after feeding diet supplemented with 8 g kg^−1^ of astaxanthin to yellow catfish (*Pelteobagrus fulvidraco*), which resulted in an increase in SOD and HSP70 activity, and a reduction in both ALT and AST, as well as an increased survival following stress and infectious challenge with *Proteus mirabilis* (Liu *et al*., 2016).

Another example of compounds with antioxidant activity is malondialdehyde (MDA, Fig. 2). Feeding of lambs with *A. platensis* (incorporated at 0.1 g kg^−1^ of food) resulted in a decrease of the animals´ serum concentrations of MDA (from 99 to 16 nmol ml^−1^ in the animals receiving the control and supplemented feed respectively). At the same time, an increase in both vitamin A (from 690 to 710 ng ml^−1^) and glutathione (from 90 to 140 ng ml^−1^) was recorded in the animals' sera (EL‐Sabagh *et al*., 2014). The same authors further reported reduction in the sera's alanine and aspartate aminotransferase, which is consistent with reduced oxidative stress to the liver. Diet supplemented with *A. platensis* (at doses ranging from 25 to 100 g kg^−1^ of feed) fed to the fish *O. mykiss* was correlated with an increase in the serum antioxidant activity (at doses ranging from 50 to 100 g kg^−1^ of feed) alongside an increased expression of the superoxide dismutase and catalase genes in the liver of the fish, when given at doses of 75 or 100 g kg^−1^ of feed (Teimouri *et al*., 2019). Feeding of the fruit fly *Drosophila melanogaster* with *Chlorella sorokiniana* (incorporated at doses of 2 or 4 mg ml^−1^) resulted in an increased expression of *SOD1*, a superoxide dismutase encoding gene, as well as resistance against H_2_O_2_‐induced oxidative stress (Qiu *et al*., 2020). Phycobiliproteins have also demonstrated potent antioxidant capacities and promoted the elimination of reactive oxygen species and increasing the concentration of anti‐oxidative enzymes (Li *et al*., 2019). Future analyses could be more investigated for bioinformatical analysis of algae and their microbiomes. So far, data sets published at IMG/MER revealed genes coding for SOD, catalases and rhodanese‐related sulfurtransferases (Table 1). Furthermore, the studying of algal genomes (*C. variabilis* NC64A and *C. subellipsoidea* C‐169) demonstrated the presence of genes coding for the biosynthesis of known antioxidants, such as chlorophyll, carotenoid, lutein and astaxanthin. These genomes are available under the accession number IMG ID 2507525016 (Table 1).

### Anti‐inflammatory and anti‐cancer properties

Sterols represent a subgroup of steroid molecules and are widespread in the cell membranes of eukaryotic organisms. Figure 2 shows the molecular structures of saringosterol and phytosterols. Various sterols have been linked to diverse health benefits, including anti‐inflammatory and anti‐cancer properties. Phytosterols from *H. pluvialis* have been shown to have cytotoxic effects on human IMR‐32 neuroblastoma cells, with a dose of 100 or 200 μM inhibiting neuronal activity by about 60% (Bilbao *et al*., 2016). Sanjeewa *et al*. (2016) have also reported anti‐inflammatory effect of the hexanoic fraction of extracts from *Nannochloropsis oculata*, suppressing nitric oxide production in LPS activated macrophages when applied at doses of 6.25, 12.5 or 25 μg ml^−1^. This fraction also showed anti‐proliferative and pro‐apoptotic effects, when applied at doses of 25 μg ml^−1^, in several human cancer cell lines.

Sterols are mostly employed in cardiovascular health, because of their ability to hinder cholesterol adsorption in the intestine (Ostlund *et al*., 2003). The saringosterol from the kelp species *Lessonia nigrescens* has been shown to have antimicrobial activity on *Mycobacterium tuberculosis* with MIC values equivalent to rifampin at 0.25 μg ml^−1^ (Wächter *et al*., 2001). Interestingly, it has been estimated that the current source of phytosterols will be unable to meet demands by 2030 (Randhir *et al*., 2020). The presence of a high variety of sterols is well established in eukaryotic algae and, more controversially, also in cyanobacteria (Volkman, 2003), although the subject has received very little considerations and much remains to be investigated in this field (Randhir *et al*., 2020).

### Immune promoters and immunomodulatory activity

A large number of plants and microorganisms are known to possess an immunostimulatory activity (Riccio and Lauritano, 2020), although the mechanisms through which microalgae cultures exert this immunostimulatory effect, often remain to be clarified. However, microalgal products have been known to induce the expression of various immune genes. Moreover, Xu *et al*. (2014) have reported an increase in digestive enzyme and an improvement in the growth performance of gibel carp (*Carassius auratus gibelio*) fed dried powder of *Chlorella* sp. (incorporated at doses as low as 4 g kg^−1^ of food). Similarly, Adel *et al*. (2016) reported a significant increase in protease activity as well as the population of lactic acid bacteria in the intestine of sturgeons (*Huso huso*) fed *A. platensis* at dose of 50 or 100 g kg^−1^ of food. These results suggested that an improvement in the digestive health of the fish, possibly linked to a prebiotic effect of the algae, may have contributed to the improved immune parameters reported in the studies. For example, polysaccharides from *Chlorella vulgaris* have been shown to promote the transcription of nitric oxide, prostaglandin E2, TNF‐α, IL‐6 and IL‐10, as well as promote cell proliferation in the murine macrophage cell line RAW264.7 (Tabarsa *et al*., 2015). Feeding of *Nile tilapia* (*Oreochromis niloticus*) with feed supplemented with 50 mg kg^−1^ of either β‐Carotene or phycocyanin‐supplemented feed resulted in a significant elevation of the activity of multiple blood immune parameters (phagocytic and lysozyme activity, immunoglobulin M levels), while expression of the genes coding for the interferon gamma and interleukin 1β was upregulated (Hassaan *et al*., 2021). Addition of dry powder from *C. vulgaris* to the diet of the Koi carp *Cyprinus carpio* at doses ranging from 50 to 100 g kg^−1^ of feed resulted in the proliferation of red and white blood cells, while inclusion of the algae at doses ranging from 20 to 100 g kg^−1^ of feed increased lysozyme activity (Khani *et al*., 2017).

Reports on supplementing feed for rainbow trout (*Oncorhynchus mykiss*) with β‐carotene rich extracts from the marine phytoplankton *Dunaliella salina* at doses of 100 to 200 mg kg^−1^ resulted in an increase in the phagocytic rate and the serum complement and lysozyme activity in the fish (Amar *et al*., 2004). In shrimp, it has been reported that feed supplementation with 3 g kg^−1^ of *Arthrospira platensis* resulted in an improvement of the phagocytic activity of haemocytes from the banana shrimp *Penaeus merguiensis*, as well as resistance to infection by *Vibrio harveyi* (Lee *et al*., 2003). In mammals, diet supplementation with extracts from *A. platensis* was found to increase the levels of IgG1 in the serum and IgA in the intestine, alongside the antibody produced in the supernatants of lymphoid cell cultures from the spleens and mesenteric lymph nodes of *A. platensis*‐fed mice (Hayashi *et al*., 1998). Interestingly, the effect was class‐specific as IgE levels were unaffected by this feed supplement. Comparable results were obtained by dogs where supplementation of the diet with 0.2% of spray‐dried *A. platensis* resulted in an increase in the levels of serum antibodies and faecal IgA with the following vaccination with a commercial anti‐rabies vaccines (Satyaraj *et al*., 2021). Investigations using *Dunaliella tertiolecta* found that extracts and purified sterols (at concentrations of 0.4 mg ml^−1^ and 0.8 mg ml^−1^ diluted 1 in 3) from this microalgae had anti‐inflammatory effects in sheep, reducing proliferation of peripheral blood mononuclear cells as well as the production of interleukin‐6, which was the opposite to what Tabarsa *et al*. (2015) reported in *C. vulgaris*, while promoting secretion of IL‐10 (Caroprese *et al*., 2012). Administration of 50 mL a day of warm‐water extracts from *A. platensis* to sheep was reported to induce secretion interleukin 12 subunit beta (IL12 p40) by peripheral blood mononuclear cells as well as the secretion of interferon‐gamma and the cytotoxic activity of activated NK cells (Hirahashi *et al*., 2002). However, diet supplemented with increasing doses of *A. platensis* (ranging from 5 to 20 g kg^−1^) resulted a dose‐dependent increase in harvested macrophages, with a higher percentage of macrophages phagocytosing sheep red blood cells (SRBC) and a higher average number of SRBC in each macrophage (Al‐Batshan *et al*., 2001). The authors of this study also reported a significant increase in nitric‐oxide production in macrophage stimulated with bacterial LPS.

### Prebiotic activity

The indigenous microbiota of microalgae represent an early and important barrier to infection. Local bacteria can inhibit bacterial infections either directly through the secretion of antimicrobial or antiviral compounds, or by competing with them for nutrient and attachment sites, a phenomenon known as competitive exclusion (Irianto and Austin, 2002; Ghanei‐Motlagh *et al*., 2021a, 2021b). Consequently, research has been performed on the possibility to defend against infection either through the direct ingestion of beneficial microorganisms (probiotic treatments) or the ingestion of substances that promote the growth of beneficial bacteria (prebiotic treatments). Several microalgae have been shown to have prebiotic activity, for example, *C. vulgaris* and *A. platensis* are known to increase the viability and survival of multiple beneficial bacteria such as lactobacilli and bifidobacteria when incorporated at doses ranging from 0.25 to 1.00% (Beheshtipour *et al*., 2012). Moreover, co‐culture with *C. vulgaris* or *Nannochloropsis oculata* has been shown to improve the antimicrobial activity of *Sulfitobacter* spp. or *Roseobacter* sp., respectively, against *Vibrio anguillarum* (Sharifah and Eguchi, 2011, 2012). More recently, it has been reported that supplementation of dogs' diets with 2 g kg^−1^ spray‐dried *A. platensis* improved the stability of the gut microbiota in dogs during periods of physical exercises (Satyaraj *et al*., 2021). It has been suggested that some algae have the opposite effect, such as sequestering valuable nutrients and reducing their availability to bacteria.

## Discussion and limitations

### Compound production and toxic compounds

The production of compounds from microalgae microbiomes is complicated by the fact that their production is often strongly influenced by the culture conditions of the algae and that these conditions are not always known for all algal strains (Abu‐Ghannam and Rajauria, 2013; Fatma, 2009). This is further complicated by different culture conditions that may affect various beneficial factors in different ways. For example, cultivation in Zarrouk medium improved the production of β‐carotene and the antioxidant properties of several strains of *Arthrospira* spp. (several dozen times for some strains) while a medium deprived of some mineral ingredients, RM6, allowed for an improved production of phycobiliproteins (Tarko *et al*., 2012). A strong seasonal effect has also been reported, although this may simply be a side‐effect of changes in light and temperature conditions (Abu‐Ghannam and Rajauria, 2013). Changes in light intensity to intensities inducing light stress have been shown to increase production of triacylglycerol by 250% and sterols by 1200% in *H. pluvialis* (Bilbao *et al*., 2016).

Optimal culture conditions for the algae will often be different from the conditions for the optimal production of the compounds of interest, as is the case for the production of carotene or antimicrobial fatty acids (Ruffell *et al*., 2016; Molino *et al*., 2018; Kaha *et al*., 2021). As one could expect, protective secondary metabolites are often produced in response to stressors which will impair algal growth (Little *et al*., 2021). However, there is no universal rule correlating harsher culture conditions with the accumulation of beneficial compounds. For example, Ru *et al*. (2020) have reported that poor growth conditions lead to an increase in the starch content of *C. vulgaris* (Ru *et al*., 2020). Even within the same species, there can be considerable differences between strains, and it will be necessary to confirm that the strain does produce the compound of interest in high quantities under the expected culture conditions (Tarko *et al*., 2012).

Several microalgae are known to secrete phycotoxins. In particular dinoflagellates are known as a major source of toxins in the marine environment (Wang, 2008). These toxins, including the alkaloid saxitoxin that is considered the most toxic among them, have been associated with neurotoxicity. Dinoflagellates toxins are normally present at relatively low levels in the environment, although this level increases during algal blooms involving these species and have been correlated with mortality events in aquatic life, for example, *Alexandrium tamarense* has been associated with mass mortalities in fish, birds and aquatic mammals in the Saint Lawrence Estuary in Canada (Starr *et al*., 2017). These toxins are known to bioaccumulate along the food chain with predatory carnivorous fish harbouring higher levels of the toxins, and diseases in humans are often associated with the consumption of contaminated seafood. In the case of ciguatera, caused by ciguatoxin and maitotoxin produced by microalgae of the genus *Gambierdiscus*, cases are more generally associated with the consumption of contaminated reef‐dwelling fish. The presence of these toxins is likely a major reason why dinoflagellates have received less attention as a source of health products (Friedman *et al*., 2017).

Cyanobacteria are known to secrete a large number of various toxins with hepatotoxic effects as well as known neurotoxins such as kalkitoxin and saxitoxin, and more than 100 species of cyanobacteria have been shown to secrete toxins (Singh *et al*., 2005; Sacilotto Detoni *et al*., 2016; Zerrifi *et al*., 2021). Extracts from the marine cyanobacterium *Trichodesmium erythraeum* from microalgal blooms on the Brazilian coastline were found to contain microcystins, cylindrospermopsins and saxitoxins, and showed toxic antimitotic activity against larvae of the green sea urchin (*Lytechinus variegatus*) but not against mice (Proença *et al*., 2009). Saxitoxins from *T. erythraeum* were also connected to mortality events in farmed pearl oysters (Negri *et al*., 2004). Some of these cytotoxic activities may prove beneficial, for example, in the development and antitumor therapeutants; however, they will make commercial adoption of microalgae more complex (Parra‐Riofrío *et al*., 2020).

### Sustainability – effect of environmental conditions and costs

From a sustainability perspective, microalgae production allows to capture CO_2_ with higher average bioenergetic yield on sunlight than higher plants: 10% vs. 5% respectively (Williams and Laurens, 2010). Such higher yield allows to reduce the use of land for cultivation. While closed photobioreactors enable higher productivity and finer control of growth conditions than open pond systems, they also display higher energy consumptions (Brentner *et al*., 2011; Valdovinos‐García *et al*., 2021). Recent Life Cycle Assessments (LCA) studies of microalgae cultivation in closed reactors (Pérez‐lópez *et al*., 2014a; 2014b; Porcelli *et al*., 2020) showed that in pilot scale astaxanthin production from *Haematococcus pluvialis* and in the production of *Tetraselmis suecica* and *Phaeodactylum tricornutum*, the main contributor to most environmental impact categories were by far the electricity consumption during the cultivation stage. In these cultivation systems, electricity is mainly consumed for mixing and pumping large water volumes and for lighting the reactor when necessary. Regarding outdoor cultivation, the need to thermoregulate the system is one of the main drivers for electricity consumption (Pérez‐López *et al*., 2017; Smetana *et al*., 2017; Schade and Meier, 2019; Duran Quintero *et al*., 2021). A common insight from these studies is that the environmental performance of cultivating a specific strain of microalgae is highly dependent on the location, cultivation period and suitable thermal range of the strain (Duran Quintero *et al*., 2021).

Microalgae are becoming increasingly interesting for the extraction of high value compounds, rather than as feedstock for the refining of low value products such as biofuels. Concerning this extraction, Pérez‐lópez *et al*. (2014a, 2014b) showed that the extraction stage (methanol and KOH solutions) for the production of PUFAs, α‐tocopherol, chlorophyll, β‐carotenoid and polyphenols by *Tetraselmis suecica* was the second most important contributor to most environmental impact categories, but remained far behind the cultivation stage. Supercritical CO_2_ fluid extraction was used for astaxanthin extraction Pérez‐lópez *et al*. (2014a, 2014b) and accounted for less than 10% of the considered impact categories. Overall, even if the extraction method depends on the targeted bioactive compound, cultivation will remain the main environmental hotspot for new microalgal strains. Crucial to limit the environmental impacts of a microalgal production is the valorization of coproducts within an integrated biorefinery approach (Da Silva *et al*., 2014; ‘t Lam et al., 2018). Microalgae residual biomass can serve as substrate for biogas production via anaerobic digestion and the residual digestate can substitute the production of fertilizers (Collet *et al*., 2011; Pérez‐lópez *et al*., 2014a, 2014b). Depending on the nutrient profile of the biomass, it could also be used to substitute animal feed (Draganovic, 2013). Due to the high diversity of the assumptions, parameters and production technologies, for strains that have not been cultivated yet, it is worth noting that their behaviour, optimal growth conditions and productivity in given reactors and locations are currently difficult to anticipate and therefore highly uncertain (Mata *et al*., 2010; Barra *et al*., 2014).

The potential need to induce bioactive molecule production by specific cultivation conditions, such as high‐intensity lighting for astaxanthin production from *Haematococcus pluvialis*, could greatly affect the final environmental impacts (Pérez‐lópez *et al*., 2014a; 2014b; Onorato and Rösch, 2020). Ultimately, the overall sustainability of producing microalgae‐based bioactive molecules depends on the final efficiency and quality of the produced compounds. Finding synergistic products would therefore be of great interest. Indeed, the compound's capacity to substitute alternatives, tackle key issues such as fish farming health management and the needed doses will highly affect its environmental performance (Liu *et al*., 2016; Lieke *et al*., 2020).

### Opportunities for future research

Currently, the main knowledge gap is linked to the correspondingly low numbers of algal species studied, as only a small percentage of species have been investigated compared to the large number of microalgal species that exist (Connelly, 2014), as illustrated by the number of times the same genus names are repeated in the present review. This is especially true when taking into account the strain differences previously mentioned. Therefore, one of the most urgent tasks is to increase our knowledge by investigating new microalgal species, and focusing on other than well‐known genera (Yarnold *et al*., 2019).

Optimal culture conditions are not always known for all algal strains, and neither are the optimal conditions for the production of the compounds of interest. This means that additional research is needed for each individual strain to optimize the farming protocol for both biomass and the production of the compounds of interest (Ruffell *et al*., 2016).

Another approach is to identify or create mutants that overexpress the compounds of interest. Genetic engineering of microalgae is shown to be possible, although it is more technically difficult than for other organisms (Qin *et al*., 2012; Spicer and Purton, 2017; Charoonnart *et al*., 2018).

Consequently, alternative and potentially more promising pathways could be integrated in multi‐omics approaches to stimulate production within microalgae and their microbiota, and transfer and expression of biosynthetic pathways in suitable heterologous host species and strains. (Meta)genomics, (meta)transcriptomics, (meta)proteomics and MS‐based metabolomics approaches can thereby help unravelling biosynthetic pathway activity constraints in microalgae and their microbiomes and identifying beneficial chemical compounds, that is, elicitors, that can be used to stimulate the production of compounds of interest (Maghembe *et al*., 2020). Such approaches in combination with advanced bioinformatics assessment of (meta)genomic contents, for example, using tools like antiSMASH and MiBIG (Kautsar *et al*., 2020; Blin *et al*., 2021) can also help identifying the boundaries if biosynthetic gene clusters (BGCs) that encode the biosynthetic machinery for such compounds (Table 1). Combining this knowledge with advanced long‐insert cloning technologies and transfer to a panel of heterologous expression hosts can yield production of the compound of interest in a system that is better accessible to genetic modification for the purpose of optimizing production and further compound engineering (Nah *et al*., 2017; Ke and Yoshikuni, 2020). Such techniques may also be helpful in reducing the presence of detrimental compounds, for example, toxic compounds, produced by these algae.

## Conclusions

Microalgae in combination with their associated microbiota are very promising as health management tools: not only have several species shown potential, harbouring antimicrobial, immune‐stimulating and antioxidant substances but also because of their variety and the relatively small numbers that have been investigated, it is likely that many useful compounds remain to be discovered. In this context, much research remains to be performed to identify new compounds. In addition, we have to clarify their mechanisms of action and make their application practical, by optimizing production methods and reducing their costs of production as well as multi‐omics approaches.

## Conflict of interest

None declared.

## Authors’ contributions

IK, SML, GH, YA, PJ, JLN, MP, AW and WRS contributed to general concept and design and writing of the article. All authors contributed to manuscript revision, read and approved the submitted version.
